# Hantavirus seroprevalence and associated factors for exposure in south-central Uganda  

**DOI:** 10.1080/22221751.2026.2665002

**Published:** 2026-05-24

**Authors:** Gerald Katushabe, Victor Ssempijja, Deepashri Rao, Josephine Nalwadda, Kyle Rosenke, Steven J. Reynolds, Jonah Omooja, Irene Andia Biraro, Mary K. Grabowski, Evan A. Mihalakakos, Ronald M. Galiwango, Robert Ssekubugu, Denis K. Byarugaba, Heinz Feldmann, David W. Hawman

**Affiliations:** a Laboratory of Virology, NIAID/NIH International Centers for Excellence in Research, UVRI, Entebbe, Uganda; b College of Health Sciences, School of Medicine, Clinical Epidemiology Unit, Makerere University, Kampala, Uganda; c Clinical Monitoring Research Program Directorate, Frederick National Laboratory for Cancer Research, Frederick, MD, USA; d Laboratory of Virology, Division of Intramural Research, NIAID/NIH, Rocky Mountain Laboratories, Hamilton, MT, USA; e Rakai Health Sciences Program, Kalisizo, Uganda; f Laboratory of Immunoregulation, NIAID/NIH, Bethesda, MD, USA; g Department of Pathology, Johns Hopkins School of Medicine, Baltimore, Maryland, USA; h College of Veterinary Medicine, Makerere University, Kampala, Uganda; i Laboratory of Viral Zoonoses, Integrated Research Facility, Division of Intramural Research, NIAID/NIH, Frederick, MD, USA

**Keywords:** Orthohantavirus, seroprevalence, rodents, antibodies, Uganda, Africa

## Abstract

Orthohantaviruses are largely rodent-borne pathogens that can cause haemorrhagic fever with renal syndrome and hantavirus cardiopulmonary syndrome. In Uganda, the risk of human exposure is heightened by known rodent hosts, close human-rodent interaction in rural areas, and poor housing conditions. Despite this risk, data on human exposure remain scarce. This study sought to ascertain the seroprevalence of orthohantavirus exposure and identify associated factors with exposure among residents of the greater Masaka-Rakai region in Uganda. Seropositivity was assessed for orthohantavirus-specific IgG antibodies using commercial enzyme-linked immunosorbent assays. Logistic regression models were used to identify factors associated with seropositivity. Among 1,199 sera samples, orthohantavirus population-weighted seroprevalence was 7.4% (95% CI: 3.91–10.80). Males had a higher seroprevalence, while higher socioeconomic status was associated with a reduced burden of exposure to orthohantavirus. This study reports evidence of orthohantavirus exposure in Uganda, highlighting a previously underrecognized zoonotic risk in the region likely driven by close contact with rodent reservoirs and poor living conditions. The higher burden among males and lower-burden association with higher socioeconomic status, highlights the need for improved housing, rodent control, and integration of orthohantavirus surveillance into national public health programmes.

## Introduction

Orthohantaviruses are zoonotic pathogens belonging to the *Hantaviridae* family in the *Elliovirales order, Bunyaviricetes class*. They are enveloped viruses with a tri-segmented, single-stranded, negative-sense RNA genome [1–4]. These viruses are mainly transmitted to humans through aerosolized excreta from infected rodents, although shrews and bats may also act as reservoirs [5–9]. Hantaviruses in the genus *Orthohantavirus* [10] are responsible for two major clinical syndromes: haemorrhagic fever with renal syndrome (HFRS) in Europe and Asia and hantavirus cardiopulmonary syndrome (HCPS) in the Americas [11]. These syndromes can range from subclinical or mild febrile illness to severe multisystem failure with case fatality rates of up to 50% in HCPS and up to 12% in HFRS [12–16].

Globally, more than 200,000 orthohantavirus-related infections occur annually, with pooled seroprevalence estimates of 6.84% in Asia, 2.98% in Europe, 2.34% in the Americas, and 2.21% in Africa [17–19]. This burden is likely underestimated, especially in low-resource settings with limited diagnostic capacity. Treatment is mainly supportive; ribavirin may help if given early in HFRS, but no licensed antiviral exists [20–22]. Vaccines are not widely available; only Hantavax™, a regionally licensed inactivated vaccine used in South Korea for HFRS caused by Hantaan and Seoul viruses, is in use. Its efficacy remains uncertain, though it shows moderate effectiveness in high-risk populations [22–25].

According to the World Health Organization, human infections commonly occur in rural areas where people are exposed to infected sylvatic rodents in settings such as farms and fields [26]. Serological evidence of orthohantavirus exposure has been reported in various sub-Saharan African countries, including Guinea, Kenya, South Africa, and Madagascar, with prevalence rates ranging from 1.0% to 12% [17,27–32]. In south-central Uganda, particularly the greater Masaka-Rakai region, subsistence farming and poor housing conditions increase human interaction with potential orthohantavirus reservoirs such as rodents, heightening the risk of transmission. Despite these factors and known orthohantavirus reservoirs, such as *Mastomys natalensis* and *Rattus norvegicus* [33,34], no ecological or epidemiological data exist for Uganda. This study aimed to estimate the seroprevalence of orthohantavirus infections and identify associated factors of exposure among residents in the Rakai Community Cohort.

## Materials and methods

### Study setting and population

This study was undertaken using retrospective samples from the Rakai Community Cohort Study (RCCS), ongoing since 1994 by the Rakai Health Sciences Program (RHSP), a longitudinal population-based cohort of ∼20,000 individuals aged 15–49 years in the greater Masaka-Rakai region of south-central Uganda [35,36]. At ∼18-month intervals, RCCS holds a census of all residents, whether permanent or transient, in every household from 41 agrarian, trading, and fishing cohort communities. Structured confidential interviews are conducted with consenting individuals aged 15–49 years old, and a blood sample, GPS coordinates, and sociodemographic and health data are collected.

### Study design, sampling strategy and demographics

**Figure 1.**
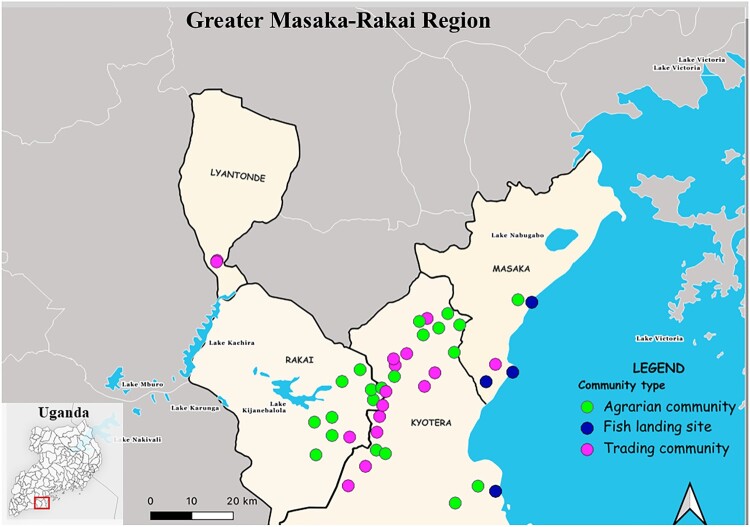
Map of greater Masaka – Rakai region in south-central Uganda, showing the location of study site communities. Community types are represented using a blue decagon (fish landing sites), green decagon (agrarian communities), and purple decagon (trading communities). The bottom-left inset shows a map of Uganda with the south-central region highlighted. The map was modified and adapted from Mihalakakos et al.

Our sample set has been previously described and evaluated for Crimean-Congo hemorrhagic fever virus seropositivity [37]. This was a cross-sectional study nested within the RCCS cohort, using retrospective samples and data collected from June 2018 to October 2020. The 1,199 study participants came from 21 agrarian communities (n = 400), 16 trading communities (n = 399), and 4 Lake Victoria fishing communities (n = 400) in south-central Uganda (Figure 1). As previously published, agrarian, trading and fishing communities differed by all demographic characteristics, including age, gender, education level, occupation, and socioeconomic status (provided in Supplemental Table 1 for reference).

### Laboratory testing for orthohantavirus exposure

A two-stage serial antibody testing algorithm was used to test for exposure to orthohantaviruses. First, serum samples were heat-inactivated at 56°C for 1 h, then screened for orthohantavirus IgG antibodies, using the AVIVA Systems Biology commercial IgG ELISA kit (OKNA00138), following the manufacturer’s instructions. The kit supplier reports a sensitivity of 98.3% and a specificity of >95.9% for this assay. Thereafter, positive samples by the AVIVA ELISA were tested with the EUROIMMUN Anti-Hanta Virus Pool 1 “Eurasia” ELISA (IgG), following the manufacturer’s instructions. The EUROIMMUN kit reported a sensitivity of 88.2–100% and a specificity of 94.1–100%, depending on the study setting and reference standard. A sample was considered positive only if it tested positive on both assays. Optical densities were measured using the Accuris™ SmartReader™ 96 Absorbance Plate Reader model 9600.

### Statistical and data analysis

**Figure 2.**
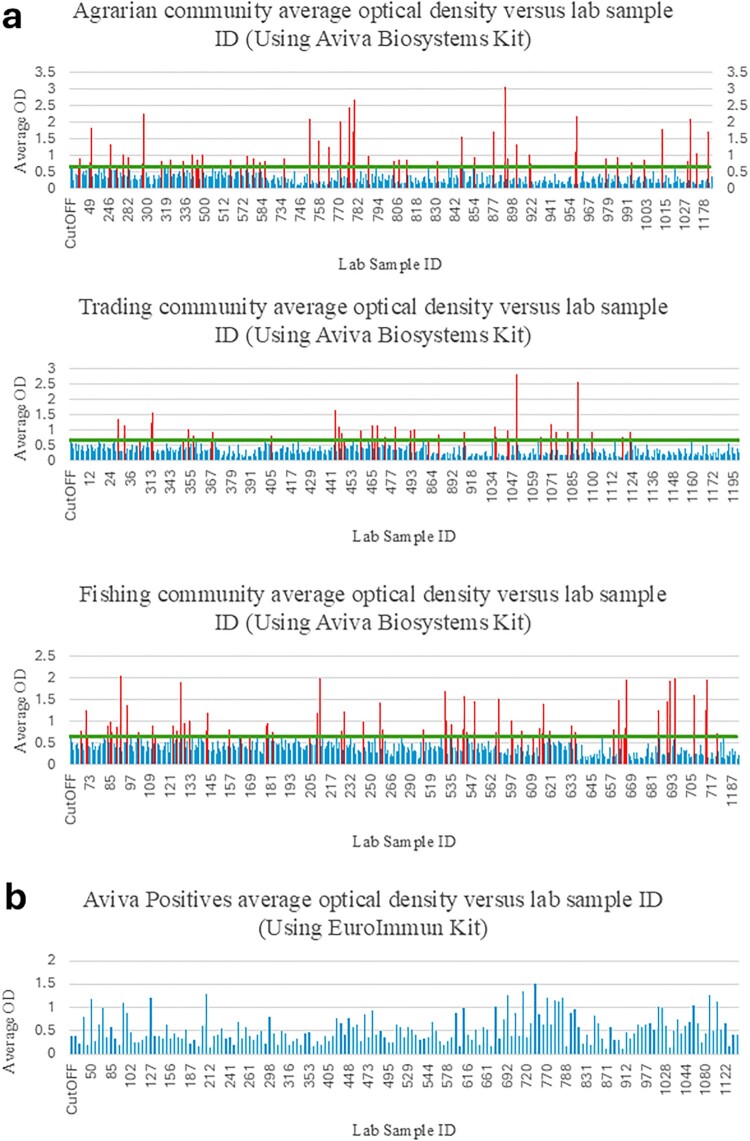
Optical densities of serum samples evaluated by two commercial orthohantavirus assays. (a) Serum samples from agrarian, trading and fishing communities were initially screened by the Aviva Systems Biology human hantavirus IgG kit according to the provided protocol. The green line indicates the absorbance cutoff for positive samples as determined by the multi-plate average of the kit-provided cutoff sample. Presumptive positive samples were then confirmed by the EuroImmun Anti-Hanta Virus Pool 1 Eurasia ELISA kit, according to instructions (b). Only samples positive by both assays were considered positive for further statistical analyses.

Frequencies and percentages were used to describe the distributions of potential confounding variables, and chi-square or Fisher’s exact tests were used to test differences in distributions between the community types. Potential confounders included sex, age, education, occupation, social economic status (SES), ownership of cultivatable land and animal ownership. SES was determined using principal component analysis of household assets, and then participants were divided into 4 strata to maximize within-group homogeneity, such as grouping together participants with the same weight. The strata were categorized progressively as lowest, low-middle, high-middle, and highest SES (provided in Supplementary table 2). The primary outcome was the seroprevalence of orthohantaviruses, estimated as the proportion of individuals in the selected communities of south-central Uganda who tested positive for orthohantavirus-specific IgG antibodies via ELISA. The community type seroprevalence was computed as the proportion of positive samples with a 95% confidence interval (CI), and overall seroprevalence was computed from the population-weighted proportion of orthohantavirus positive samples with their corresponding 95% CIs. Differences in seroprevalence across community type were assessed using Chi-square or Fisher’s exact tests**.** A significant association between potential confounders and the prevalence level of orthohantavirus was assessed using odds ratios with corresponding 95% CIs that were computed from bivariable and multivariable logistic regression analysis. Potential confounders with *p* < 0.2 in the bivariable analysis were included in the multivariable model; significance was set at *p* < 0.05. Variables with *p*-value < 0.2 in bivariate analysis and known confounders were included in multivariable logistic regression, after checking for multicollinearity using the variance inflation factor (VIF) and outliers using Leverage and influence diagnostics and Dbeta values**.** Model fit was assessed using the Hosmer-Lemeshow test. Data analysis was done using STATA version 18.5 Standard Edition software [38]. Assay absorbance values presented in Figure 2 were graphed using Microsoft Excel.

### Geospatial mapping of the studied communities

The map was generated in QGIS version 3.40 “Bratislava” [39] to depict the studied RCCS communities (Figure 1). The GPS coordinates of RCCS from 41 communities sampled between June 2018 and October 2020 were utilized to create the map. Community type was represented using distinct colours.

### Ethical consideration

The Rakai Community Cohort (parent study) was approved by the Research and Ethics Committee of the Uganda Virus Research Institute (GC/127/19/03/709), the Uganda National Council for Science and Technology (HS-364), and the John Hopkins School of Medicine Institutional Review Board (IRB00204691). All participants signed written informed consent prior to enrollment that allowed for future anonymized sample testing. Participant data was de-identified before data analysis.

### Data sharing

The data used in this study can be requested from datarequests@rhsp.org.

## Results

### Laboratory serology test results

Among the 1,199 samples, 168 tested seropositive for orthohantavirus under the AVIVA Biology Systems commercial IgG ELISA kit, and 88 of these positives tested seropositive under EUROIMMUN Anti-Hanta Virus Pool 1 “Eurasia” ELISA (IgG) kit (Figure 2). As the species of orthohantaviruses in Uganda are unknown, the discordance between positives in the first test and positives in the second test may be due to differences in the antigen preparations and their sensitivity to detect responses against Ugandan orthohantaviruses. Nevertheless, we elected to consider only individuals positive by both assays as positives for further analysis.

### Orthohantavirus seroprevalence by community type

Among the 1,199 samples, we found an average seropositivity of 7.8% (95%CI: 5.5-10.8) in fishing communities, 8.3% (95%CI: 5.9-11.4) in agrarian communities, and 6.0% (95%CI: 4.1- 8.8) in trading communities for an overall population-weighted seroprevalence of 7.4% (95%CI: 3.91-10.80). Prevalence of orthohantaviruses was similar in all three communities (*p* = 0.446) (Table 1).

**Table 1.** Community-level of orthohantavirus exposure.

		Community type		Overall
Exposure Variable	Agrarian	Trading	Fishing	
Orthohantavirus serology status				
No	367(91.7%)	375(94.0%)	369(92.2%)	1111(92.7%)
Yes	33(8.3%)	24(6.0%)	31(7.8%)	88(7.3%)
Chi-squared *p*-values				
Agrarian vs trading vs fishing	0.446			

### Factors associated with orthohantavirus exposure

In multivariable analysis, male sex was significantly associated with higher seropositivity of orthohantavirus (aOR [adjusted odds ratio]: 1.80; 95%CI: 1.11–2.91; *p* = 0.016). Additionally, individuals in higher socioeconomic categories had significantly lower seropositivity compared to those in the poorest category; Low Middle (aOR: 0.46; 95%CI: 0.25–0.85; *p* = 0.014), High Middle (aOR: 0.47; 95%CI: 0.26–0.85; *p* = 0.013), and Highest (aOR: 0.46; 95%CI: 0.22–0.97; *p* = 0.041), Table 2.

**Table 2.** Factors associated with orthohantavirus exposure.

Variable	Orthohantavirus status % (n/N)	Univariate		Multivariable	
		uORs (95% CIs)	p-value	aORs (95% CIs)	p-value
Community type					
Trading	6%(24/399)	Ref		Ref	
Agrarian	8%(33/400)	1.40(0.81–2.42)	0.222	1.39(0.73–2.65)	0.315
Fishing	8%(31/400)	1.31(0.76–2.28)	0.334	1.12(0.56–2.23)	0.752
Sex	** **				
Female	5%(32/607)	Ref		Ref	
Male	9%(56/592)	**1.88**(**1.20 - 2.94)**	**0**.**006**	**1.80**(**1.11–2.91)**	**0**.**016**
Age (years)	** **				
15–24	8%(34/449)	Ref		Ref	
25–34	5%(17/378)	0.57(0.32 - 1.05)	0.070	0.54(0.28–1.00)	0.051
>35	10%(37/372)	1.35(0.83 - 2.19)	0.230	1.17(0.69–1.98)	0.566
Education	** **				
None	3%(1/38)	Ref			
Primary	8%(48/598)	3.23(0.43–24.05)	0.253		
Secondary/Tertiary	7%(39/563)	2.75(0.37–20.61)	0.324		
Occupation	** **				
Indoor/Formal	5%(13/266)	Ref		Ref	
Agriculture	8%(26/309)	1.79(0.67–1.91)	0.097	1.53(0.72–3.24)	0.270
Fishing	9%(11/117)	2.01(0.63–2.59)	0.099	1.36(0.46–3.82)	0.559
Commercial/Skilled labour	8%(38/507)	1.58(0.33–1.21)	0.168	1.54(0.76–3.10)	0.230
Socioeconomic status					
Lowest	12%(39/330)	Ref			
Low Middle	6%(17/289)	0.47(0.26–0.84)	**0**.**012**	0.46(0.25–0.85)	**0**.**014**
High Middle	6%(20/352)	0.45(0.26–0.79)	**0**.**005**	0.47(0.26–0.85)	**0**.**013**
Highest	5%(12/228)	0.41(0.21–0.81)	**0**.**010**	0.46(0.22–0.97)	**0**.**041**
Low Middle Vs High Middle**^a^**		0.96(0.50–1.88)	0.914	1.02(0.52–2.00)	0.952
Low Middle Vs Highest**^b^**		0.89(0.42–1.90)	0.761	0.96(0.45–2.08)	0.926
High Middle Vs Highest**^c^**		0.92(0.44–1.93)	0.829	0.94(0.45–1.98)	0.880
Cultivable land	** **				
No	8%(29/353)	Ref			
Yes	7%(59/846)	0.84(0.53–1.33)	0.453		
Animal ownership					
No	8%(39/471)	Ref			
Yes	7%(49/728)	0.80(0.52–1.24)	0.316		

uOR =   univariate odds ratio; aOR =   adjusted odds ratio; Ref = Reference

Commercial/Skilled labour includes trading, shopkeeping, bar and restaurant work, hairdressing, casual labour, construction, mechanics, truck driving, and boda-boda riding.

Indoor-based or formal occupations include housework, teaching, clerical work, students, police/military, and medical workers.

**^a^**Pairwise comparison of Low Middle (Reference category) and High Middle SES households; **^b^**Pairwise comparison of Low Middle (Reference category) and Highest SES households; **^c^**Pairwise comparison of High Middle (Reference category) and Highest SES households.

No statistically significant associations were observed in multivariable analysis between orthohantavirus seropositivity and community type, occupation, animal ownership, and age.

## Discussion

To our knowledge, our study is the first to report orthohantavirus seroprevalence data and associated risk factors for exposure in Uganda. Our findings of orthohantavirus seroprevalence of more than 7 individuals per 100 persons in this rural setting of Uganda demonstrate substantial and previously unrecognized circulation, indicating that human exposure is not incidental but consistent with ongoing, likely endemic transmission. Given the non-specific clinical presentation of orthohantavirus infections and the lack of routine diagnostic testing, this level of exposure strongly suggests that infections are systematically underdiagnosed or misclassified among undifferentiated febrile illnesses. Accordingly, our results have immediate clinical and public health relevance, supporting the inclusion of orthohantavirus infection in differential diagnoses and providing a critical evidence base for targeted surveillance, reservoir studies, and viral characterization. Comparing with studies elsewhere, this finding is similar to that from Chile (7.5%), Gabon (8%), and Kenya (8.1%), but exceeds seroprevalences reported in the Democratic Republic of Congo (2.4%) and Côte d’Ivoire (3.9%) [27,30,40]. Differences in assay selection, antigen composition, test algorithm and confirmatory thresholds across studies may influence the outcome of studies, which are, in general, still rather consistent [30].

Male sex was significantly associated with 80% higher burden of orthohantavirus exposure compared to females, potentially due to greater involvement in outdoor occupations that increase contact with rodent-infested environments. In our study, 61% of males in the cohort reported agricultural work as their primary and/or secondary occupation, which often involves close interaction with rodent-infested fields and increases the likelihood of inhaling aerosolized rodent excreta, the principal route of orthohantavirus transmission. Men are at increased risk of orthohantavirus exposure and resulting disease because they are disproportionately engaged in high-risk occupational and recreational activities such as farming and forestry work [41,42]. These activities lead to a higher rate of diagnosed infection, a pattern observed in Finland and in studies across Europe. Analyses of notifiable infectious diseases in Germany similarly show that men are more frequently affected by rodent-borne and vector-borne infections due to higher expositional risk linked to occupational and leisure activities, reinforcing the role of behavioural and environmental factors in shaping male-biased infection patterns [43].

Participants from higher socioeconomic status had significantly lower burden of orthohantavirus exposure compared to those in the lowest group. Specifically, those in the low-middle, high-middle and highest socioeconomic groups had 53%, 55%, and 59% significantly lower burden of exposure to orthohantaviruses compared to those in the lowest SES group. This suggests that the socioeconomic gradient is driven primarily by the stark contrast between the lowest group and all higher categories, rather than by meaningful differences within the higher socioeconomic group*.* The inverse relationship may be due to the social gradient, whereby people from lower socioeconomic backgrounds are at increased risk of orthohantavirus infection, possibly explained by the distribution of housing materials. While only 5.8% of the poorest households had used modern floor construction materials (tiles, cement) and 32.4% had used modern wall construction materials (bricks, cement), these features were nearly universal in the wealthiest group. Makeshift/mud floors and mud/makeshift walls in poorer households provide favourable conditions for rodent entry, nesting, and contamination, thereby heightening the risk of orthohantavirus exposure. A potential even more important factor is hygiene, which may differ substantially across socioeconomic groups. In lower socioeconomic households, limited waste management options and easy access to food sources around the home can attract rodents and thereby increase the likelihood of human exposure. In contrast, durable flooring and wall construction among wealthier households, combined with generally better hygiene conditions that reduce rodent attractants, may act as protective barriers against rodent intrusion and contamination, helping explain the observed socioeconomic gradient in orthohantavirus infection. Similar findings were reported in a study from Amazon, Brazil, where lower socioeconomic status was associated with greater orthohantavirus seropositivity [44]. However, the standard RCCS questionnaire does not capture data on rodent exposure, and further studies will be needed to determine whether socioeconomic status correlates with rodent exposure inside and outside the home.

This cohort was previously evaluated for Crimean-Congo hemorrhagic fever virus (CCHFV) seroprevalence [37] for which we obtained an overall seroprevalence of 4.25%. In contrast with our orthohantavirus results, which showed no association between orthohantavirus seropositivity and livestock ownership, CCHFV seropositivity was associated with cow and pig ownership, consistent with the recognized role of livestock in CCHFV transmission [45,46]. Similarly, community type was significantly associated with increased CCHFV seropositivity, with fishing communities at significantly greater seroprevalence than agrarian or trading communities. In contrast, we found no association with community type and orthohantavirus exposure. The distinct vectors, ticks versus rodents, may explain the association between community type and exposure to CCHFV but not orthohantaviruses. Peridomestic rodents may make human exposure to orthohantaviruses independent of local ecology. In contrast, the Hyalomma tick vector for CCHFV depends on small and large mammalian hosts for its lifecycle, likely making CCHFV exposure more dependent on the local ecology. Nevertheless, we identified 7 individuals seropositive for both CCHFV and orthohantaviruses, of whom 5 were from fishing communities and 1 each from trading and agrarian communities. These co-exposure instances suggest an opportunity for overlapping risk mitigation strategies for zoonotic viruses in, for instance, fishing communities. However, the relative infrequency of CCHFV and orthohantavirus exposure in our cohort indicates that public health interventions will require tailoring to each specific virus for maximum public health benefit. Together, although rare, individuals with co-exposure to vector-borne diseases indicate that distinct risk factors for these vector-borne diseases overlap in Ugandan communities and warrant further investigation.

Our study was subject to a number of noteworthy limitations. First, key variables, such as rodent density, climatic conditions (e.g. rainfall, temperature, humidity), exposure to rodents or rodent droppings, which are known predictors of orthohantavirus exposure, were not part of the standard RCCS questionnaire and were therefore not assessed. Furthermore, our study analyzed serum samples exclusively from HIV-negative individuals. Given the high prevalence of HIV in Uganda, this excludes an important population of Ugandans from our analyses. Further investigation is warranted to explore whether HIV infection or conditions associated with HIV status have any relationship with orthohantavirus exposure, as HIV-associated immune suppression may increase susceptibility to infection or severe disease or may have covariate factors that influence both HIV and orthohantavirus exposure. Lastly, to our knowledge, neither of our assays have been specifically validated against African orthohantaviruses, and it is possible that these assays have reduced sensitivity or specificity for detecting responses against these viruses. Future studies identifying the orthohantaviruses circulating in Uganda and evaluating cross-reactivity of responses against related orthohantaviruses are worthwhile.

## Conclusion

In this study, about 7% of individuals had antibodies to orthohantavirus, indicating notable exposure in Rakai, Uganda. Male sex was significantly associated with a higher burden of exposure, while individuals from higher socioeconomic backgrounds had a lower burden of exposure to orthohantaviruses. These findings underscore the importance of considering gender and socioeconomic factors in orthohantavirus prevention strategies. Our findings suggest orthohantaviruses are circulating in Uganda, and increased awareness and national surveillance to better assess and monitor orthohantavirus infection/disease burden are warranted. Health promotion efforts should target high-risk populations, including men, emphasizing the use of protective gear and safe practices, as well as general rodent control efforts. Despite our data indicating orthohantaviruses are circulating in Uganda, genetic characterization of Ugandan orthohantaviruses is lacking; further research into orthohantavirus detection and characterization in the human end host and rodent reservoirs is needed to inform public health policy and strengthen diagnostic capacity and reporting.

## Acknowledgements

We are deeply grateful to all participants of the Rakai Community Cohort Study for their time and invaluable contributions. Our thanks also go to the teams at the Rocky Mountain Laboratories, NIAID/NIH, the Rakai Health Sciences Program, and the NIH laboratory in Entebbe for their unwavering support during the study. In addition, we sincerely appreciate the academic supervisors, lecturers, and colleagues in the Department of Clinical Epidemiology and Biostatistics at Makerere University for their mentorship and collaborative guidance.

## Supplementary Material

Supplementary material_Orthohantavirus.docx

NIHCoverSheetPeerRevHeinrichFeldmann.pdf
