# Supplementary material for: Hantavirus seroprevalence and associated factors for exposure in south-central Uganda
Source: Emerg Microbes Infect. 2026 May 24;15(1):2665002. doi: 10.1080/22221751.2026.2665002 (PMC13202653; doi:10.1080/22221751.2026.2665002)
Supplement: Supplementary material_Orthohantavirus.docx [file TEMI_A_2665002_SM9485.docx]

**Hantavirus seroprevalence and associated factors for exposure in south-central Uganda**

**Authors:** Gerald Katushabe^1,2^, Victor Ssempijja^3^, Deepashri Rao^1,4^, Josephine Nalwadda^1,2^, Kyle Rosenke^4^, Steven J. Reynolds^1,6,7^, Jonah Omooja^1^, Irene Andia Biraro^8^, Mary K. Grabowski^1,8^, Evan A. Mihalakakos^4^, Ronald M. Galiwango^6^, Robert Ssekubugu^6^, Denis K. Byarugaba^1,9^, Heinz Feldmann^4*^, David W. Hawman^4,10*^

**Affiliations:**

^1^Laboratory of Virology, NIAID/NIH International Centers for Excellence in Research, UVRI, Entebbe, Uganda

^2^Makerere University, College of Health Sciences, School of Medicine, Clinical Epidemiology Unit, Kampala, Uganda

^3^Clinical Monitoring Research Program Directorate, Frederick National Laboratory for Cancer Research, Frederick, MD, USA

^4^Laboratory of Virology, Division of Intramural Research, NIAID/NIH, Rocky Mountain Laboratories, Hamilton, Montana, USA

^6^Rakai Health Sciences Program, Kalisizo, Uganda

^7^Laboratory of Immunoregulation, NIAID/NIH, Bethesda, MD

^8^Department of Pathology, Johns Hopkins School of Medicine, Baltimore, Maryland, USA

^8^Makerere University, College of Health Sciences, School of Medicine, Department of Internal Medicine, Kampala, Uganda

^9^Makerere University, College of Veterinary Medicine, Kampala, Uganda

* Corresponding authors:

Heinz Feldmann E-Mail: [feldmannh@niaid.nih.gov](mailto:feldmannh@niaid.nih.gov)

David W. Hawman E-mail: [david.hawman@nih.gov](mailto:david.hawman@nih.gov)

**Supplemental material**

**Methods**

**Statistical analysis-** Household asset-based measure (ABM) of Socioeconomic status (SES) was computed based on RCCS census collected information on six household assets which included: home construction (modern materials used for the roof, walls, and floor); access to a latrine; electricity, and possession of a radio. Modern home construction materials include iron, tiles, cement, and brick as compared with traditional materials, such as mud, wattle, and thatch. Principal component analysis (PCA) was applied to determine and assign weights to household assets contributing to SES. The factor loadings indicate the relative contribution of each asset to SES. ABM score was then generated for each household and standardized using Z-scores, which were divided into quartiles representing low, low-middle, high-middle, and highest SES. Only households in which there were no missing data for any of the six asset variables were included in the analysis.

**Results**

Supplemental Table 1: **Individual demographic characteristics of RCCS round 19 participants by agrarian, trade, and fish landing site communities from June 2018–October 2020 (n=1,199).**

| Variable | | Agrarian community | | Trading community | | Fishing community | | p-value^a^ | |  |
| --- | --- | --- | --- | --- | --- | --- | --- | --- | --- | --- |
| Overall | | **400(33.4%)** | | **399(33.2%)** | | **400(33.4%)** | |  | |  |
| Gender | |  | |  | |  | |  | |  |
|  | Female | | 202(50.5%) | | 233(58.4%) | | 172(43.0%) | | **<0.001** | |
|  | Male | | 198(49.5%) | | 167(41.6%) | | 228(57.0%) | |  |  |
| Age (years) | |  | |  | |  | |  | |  |
|  | 15-24 | | 163(40.8%) | | 175(43.6%) | | 112(28.0%) | | **<0.001** | |
|  | 25-34 | | 110(27.5%) | | 112(28.1%) | | 156(39.0%) | |  |  |
|  | >35 | | 127(31.7%) | | 113(28.3%) | | 132(33.0%) | |  |  |
| Education | |  | |  | |  | |  | |  |
|  | None | | 10(2.5%) | | 7(1.7%) | | 21(5.2%) | | **<0.001** | |
|  | Primary | | 198(49.5%) | | 146(36.7%) | | 254(63.5%) | |  |  |
|  | Secondary/Tertiary | | 192(48.0%) | | 247(61.6%) | | 125(31.3%) | |  |  |
| Occupation | |  | |  | |  | |  | |  |
|  | Commercial/Skilled labour^b^ | | 131(32.8%) | | 168(42.1%) | | 208(52.0%) | | **<0.001** | |
|  | Agriculture | | 168(42.0%) | | 113(28.3%) | | 28(7.0%) | |  |  |
|  | Fishing | | 2(0.5%) | | 0(0%) | | 115(28.8%) | |  |  |
|  | Indoor/Formal^c^ | | 99(24.7%) | | 118(29.6%) | | 49(12.2%) | |  | |
| Socioeconomic status | |  | |  | |  | |  | |  |
|  | Lowest | | 82(20.5%) | | 50(12.5%) | | 198(49.5%) | | **<0.001** | |
|  | Low Middle | | 102(25.5%) | | 87(12.8%) | | 100(25.0%) | |  |  |
|  | High Middle | | 160(40.0%) | | 103(25.8%) | | 59(22.3%) | |  |  |
|  | Highest | | 56(14.0%) | | 159(39.9%) | | 13(2.2%) | |  | |
| Cultivable land ownership | |  | |  | |  | |  | |  |
|  | Yes | | 382(94.5%) | | 335(84.0%) | | 129(31.2%) | | **<0.001** | |
|  | No | | 18(5.5%) | | 64(16.0%) | | 271(67.8%) | |  |  |
| Animal ownership | |  | |  | |  | |  | |  |
|  | Yes | | 317(79.3%) | | 264(66.2%) | | 147(36.7%) | | **<0.001** | |
|  | No | | 83(20.7%) | | 135(33.8%) | | 253(63.3%) | |  |  |

*^a^Pearson’s Chi-squared test; Fisher’s exact test. Bolded variables are those with p-value <0.05.*

^b^*Commercial/skilled labour includes trading, shopkeeping, bar and restaurant work, hairdressing, casual labor, construction, mechanics, truck driving, boda-boda riding*

^c^*Indoor-based or formal occupations include housework, teaching, clerical work, students, police/military, and medical workers.*

*This cohort is the same as previously described in Mihalakakos et al* (*1*)

**Results of Asset-based measure of socioeconomic status-** The first principal component accounted for 35.3% of the total variation, indicating it is a strong single dimension of socio-economic status in this sample. The assets with the highest weights were modern wall construction (0.53), modern floor construction (0.51), electricity (0.41), latrine ownership and use (0.34), and modern roof construction (0.28). The lowest weight was for ownership of a radio (0.20) (Supplementary table 1). These weights suggest that housing quality and access to basic utilities contribute more strongly to household SES differentiation than ownership of small consumer assets. Socioeconomic status was subsequently evaluated for its association with hantavirus exposure, and the results are presented in Table 5.

Supplementary Table 2: **Component weights from principal component analysis**

| **Asset/Variable** | **Weight** |
| --- | --- |
| Modern Wall construction | 0.53 |
| Modern Floor construction | 0.51 |
| Electricity | 0.41 |
| Latrine ownership and use | 0.34 |
| Modern Roof construction | 0.28 |
| Tap water | 0.23 |
| Radio | 0.20 |
| Proportion of variance explained | 0.35 |
| Eigenvalue | 2.47 |

Supplementary Table 3: **Proportion of asset ownership Across SES categories**

| **Asset** | **Lowest(%)** | **Low-Middle(%)** | **High-Middle (%)** | **Highest (%)** |
| --- | --- | --- | --- | --- |
| Modern Floor construction | 5.8 | 79.9 | 98.3 | 100.0 |
| Modern Wall construction | 32.4 | 99.3 | 100.0 | 100.0 |
| Electricity | 14.6 | 36.7 | 90.3 | 100.0 |
| Latrine | 42.7 | 65.0 | 77.3 | 100.0 |
| Piped water | 14.6 | 19.7 | 34.1 | 100.0 |
| Modern Roof construction | 93.6 | 100.0 | 100.0 | 100.0 |

1. E. A. Mihalakakos, V. Ssempijja, R. M. Ribeiro, C. Molina-Paris, G. Katushabe, J. Nalwadda, J. Omooja, D. K. Byarugaba, K. Rosenke, S. J. Reynolds, M. K. Grabowski, R. M. Galiwango, R. Ssekubugu, H. Feldmann, D. W. Hawman, Longitudinal seroprevalence of Crimean-Congo hemorrhagic fever virus in Southern Uganda. *Emerg. Microbes Infect.* **14**, 2465315 (2025).
